# Pseudomyotonia in Romagnola cattle caused by novel *ATP2A1* mutations

**DOI:** 10.1186/1746-6148-8-186

**Published:** 2012-10-09

**Authors:** Leonardo Murgiano, Roberta Sacchetto, Stefania Testoni, Tiziano Dorotea, Francesco Mascarello, Rocco Liguori, Arcangelo Gentile, Cord Drögemüller

**Affiliations:** 1 Institute of Genetics, Vetsuisse Faculty, University of Bern, Bremgartenstrasse 109a, 3001, Bern, Switzerland; 2 Department of Comparative Biomedicine and Food Safety, University of Padua, Viale dell’Università 16, 35020, Legnaro, Italy; 3 Department of Veterinary Clinical Sciences, University of Padua, Viale dell’Università 16, 35020, Legnaro, Italy; 4 IRCCS Istituto di Scienze Neurologiche, Via Altura 3, 40139 Bologna and Department of Neurological Sciences, University of Bologna, Bologna, Italy; 5 Department of Veterinary Medical Sciences, University of Bologna, Via Tolara di Sopra 50, 40064, Ozzano Emilia, Italy

**Keywords:** Cattle, Genetic disease, *ATP2A1*, Compound heterozygous, SERCA1, Brody disease

## Abstract

**Background:**

Bovine congenital pseudomyotonia (PMT) is an impairment of muscle relaxation induced by exercise preventing animals from performing rapid movements. Forms of recessively inherited PMT have been described in different cattle breeds caused by two independent mutations in *ATP2A1* encoding a skeletal-muscle Ca^2+^-ATPase (SERCA1). We observed symptoms of congenital PMT in four related Romagnola beef cattle from Italy and evaluated SERCA1 activity and scanned *ATP2A1* for possible causative mutations.

**Results:**

We obtained four PMT affected Romagnola cattle and noted striking clinical similarities to the previously described PMT cases in other cattle breeds. The affected animals had a reduced SERCA1 activity in the sarcoplasmic reticulum. A single affected animal was homozygous for a novel complex variant in *ATP2A1* exon 8 (c.[632 G>T; 857 G>T]). Three out of four cases were compound heterozygous for the newly identified exon 8 variant and the exon 6 variant c.491 G>A(p. Arg146Gly), which has previously been shown to cause PMT in Chianina cattle. Pedigree analysis showed that the exon 8 double mutation event dates back to at least 1978. Both nucleotide substitutions are predicted to alter the SERCA1 amino acid sequence (p.[(Gly211Val; Gly284Val)]), affect highly conserved residues, in particular the actuator domain of SERCA1.

**Conclusion:**

Clinical, biochemical and DNA analyses confirmed the initial hypothesis. We provide functional and genetic evidence that one novel and one previously described *ATP2A1* mutation lead to a reduced SERCA1 activity in skeletal muscles and pseudomyotonia in affected Romagnola cattle. Selection against these mutations can now be used to eliminate the mutant alleles from the Romagnola breed.

## Background

Bovine congenital pseudomyotonia (PMT) is an impairment of muscle relaxation induced by exercise that prevents animals from performing rapid movements. So far it has been described in Italian Chianina beef cattle [1], in Belgian Blue cattle (named muscular dystonia type II) [2] and, as a single case, in a Dutch improved Red and White cross-breed calf [3]. The observed muscular stiffness in PMT is due to a delayed relaxation of muscles of the fast twitch (type II) fibers. The biochemical mechanism underlying this dysfunction is a prolonged elevation in cytoplasmic free Ca^2+^ concentration, resulting from a deficiency of the SERCA1 protein. SERCA1 is a skeletal muscle Ca^2+^-ATPase or Ca^2+^ pump which is responsible for the Ca^2+^ re-uptake into the sarcoplasmic reticulum after muscle contraction [4]. Bovine PMT disease strongly resembles the inherited Brody myopathy in humans [5], a condition of exercise-induced impairment of skeletal muscle relaxation, stiffness and cramps, caused by mutations of *ATP2A1* coding for SERCA1 [6].

In Chianina cattle, we have described PMT as autosomal recessive inherited disorder caused by a missense mutation (c.491 G>A) in exon 6 of *ATP2A1*[7]. This mutation leads to a p. Arg164His substitution in a highly conserved region of the SERCA1 protein reducing its activity [4,7]. In muscular dystonia type II affected Belgian Blue cattle, Charlier et al. [2] reported an *ATP2A1* missense mutation (c.1675C>T) leading to an amino acid substitution (p. Arg559Cys) in a highly conserved nucleotide binding domain. The same mutation was also reported to cause PMT in a single case of crossbred Dutch Improved Red cattle [3].

We observed four cases of PMT in Romagnola cattle, a white-coated Italian beef breed. The purpose of this study was to characterize the phenotype in comparison to the known PMT forms of other cattle breeds. Since we observed similar clinical signs, histological and biochemical features as in the formerly described Chianina cattle with PMT, we proceeded with a mutation analysis of *ATP2A1* and detected mutations, which most likely cause the disease in Romagnola cattle.

## Results and discussion

### Clinical features

All four affected animals (two males, two females) showed congenital exercise induced muscle contraction that prevented them from performing muscular activities of greater intensity than a simple walk at a slow pace (see Additional file 1). When startled, or forced to move faster, the muscles ‘froze up’ temporarily, inducing rigidity and uncoordinated gait without signs of pain. If these stimulations were prolonged, the muscular stiffness became so pronounced that the animals fell to the ground. After a few seconds, the muscles relaxed and the animals regained their ability to get up and move. Altogether the clinical picture strongly resembled the signs of PMT in Chianina cattle [1]. At rest electromyography (EMG) investigations of the triceps surae and gluteobiceps muscles and paraspinalis muscles of the thoracic region showed no spontaneous activity. Apart from the muscular symptoms the affected animals didn’t show any other clinical signs: They were bright, alert and in good body condition. Two of the animals (case 1 and 4) were 2 months old at the moment of the examination; the other two were 3 months old.

### Histopathology

**Figure 1:**
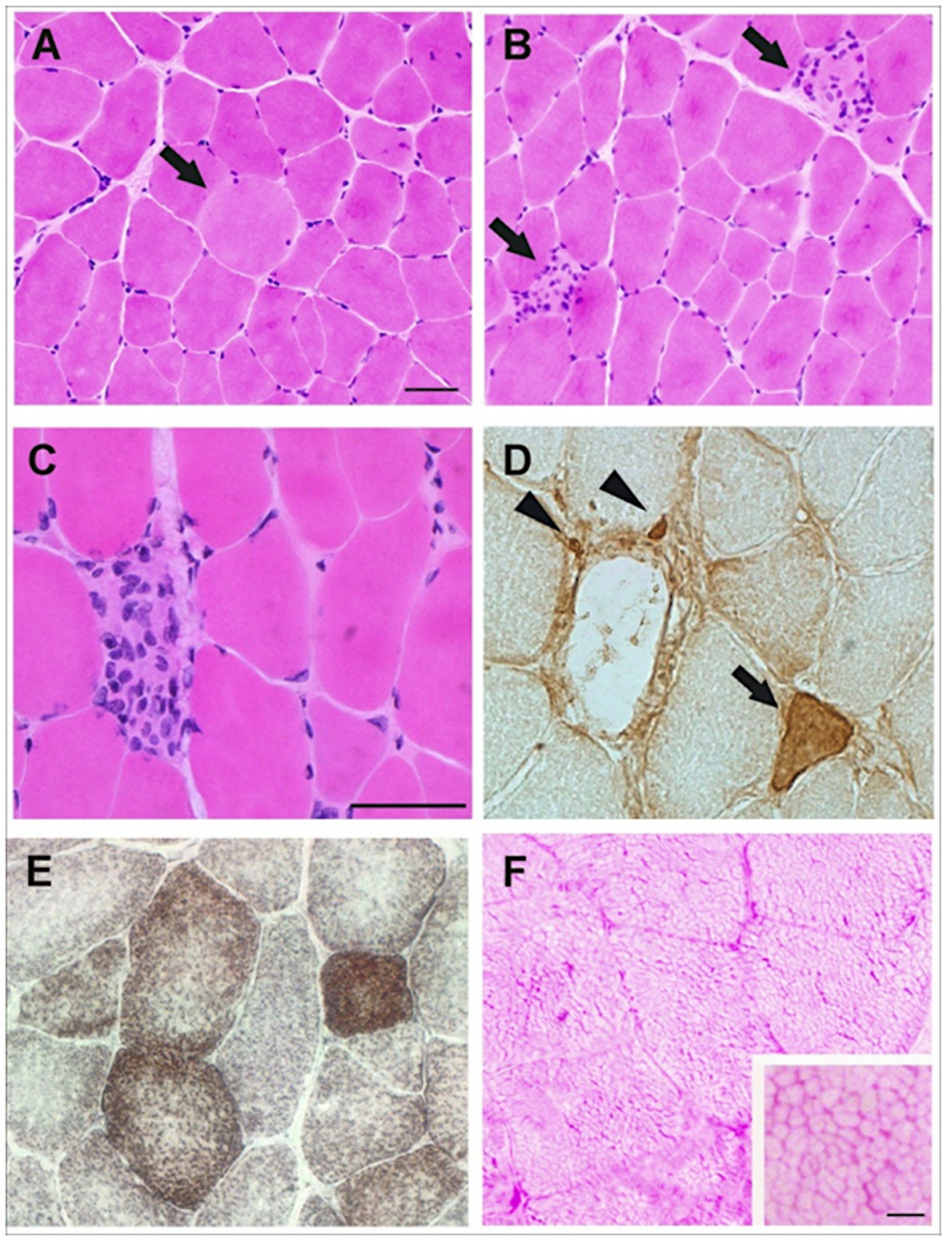
**Histopathological examination and immunohistochemistry of muscle biopsies from PMT affected Romagnola cattle.** Transversal sections from case 3 muscle biopsies were stained with H&E. A pale enlarged fiber (**A**) and pale fibers invaded by mononuclear cells (**B**) are indicated by arrows. Serial transversal sections from case 3 muscle biopsies were stained with H&E (**C**) or immunostained with anti-neonatal MHC isoform antibodies. Regenerating fibers at different stages of development (arrows) and small sized fibers (arrowheads) positive to anti neonatal MHC isoform antibodies, are indicated (**D**). Transversal sections of muscle biopsies of case 2 were stained with COX-SDH (**E**) and PAS (**F**). The polygonal honeycomb-like staining pattern is shown in more detail in the inset of panel **F**. (Scale bars: 50 μm. Scale bar inset: 5 μm).

Histopathological examination of muscle biopsies from case 2 and case 3 revealed evidence of fiber degeneration followed by fiber regeneration, more severe in case 3 than in case 2. In case 3 the cryostat sections showed enlarged fibers with pale cytoplasm (Figure 1A) and pale degenerated fibers filled with mononuclear cells (Figure 1B and 1C), many of them having an enlarged nucleus. Immunostaining of the serial sections with anti-neonatal myosin heavy chain (MHC) antibody revealed the presence of medium sized (arrow) positive fibers transiently expressing anti-neonatal MHC and small size (arrowhead) positive fibers adjacent to necrotic myofibers, indicating active fiber regeneration (Figure 1D). Histochemical analysis did not revealed variability in fiber type distribution (Figure 1E) or abnormalities in glycogen distribution (Figure 1F). The observed polygonal honeycomb-like staining pattern indicates a normal association of glycogen to sarcoplasmic reticulum membranes [8].

### Biochemical analysis

**Figure 2:**
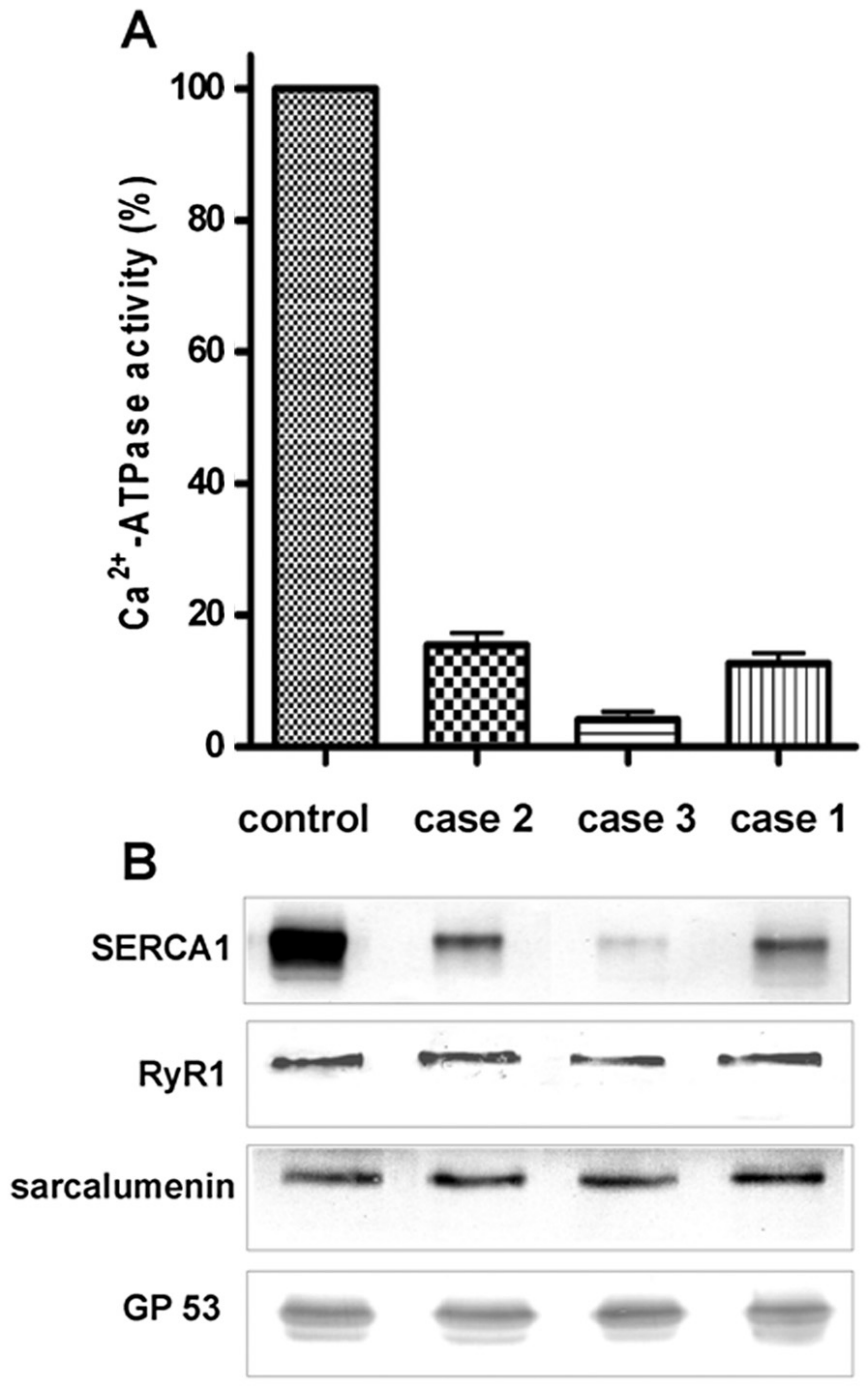
**Ca**^**2+**^**-ATPase activity and immunodetection of SERCA1 and sarcoplasmic reticulum protein markers in microsomal fractions enriched in sarcoplasmic reticulum membranes from PMT affected Romagnola cattle.** (**A**) The Ca^2+^-ATPase activity was determined by a spectrophotometric enzyme-coupled assay at optimum pCa (pCa 5) as described in Methods. Control values were obtained from different preparations of microsomes obtained from four unaffected animals of the same age. Data are expressed as the relative expression with respect to control muscle and are mean + SD. (**B**) Sarcoplasmic reticulum microsomal fractions from unaffected and PMT affected calves were separated by 5-10% SDS-PAGE and blotted onto nitrocellulose. Blots were incubated with the four indicated antibodies.

Similarities between clinical phenotypes presented here and PMT in Chianina cattle prompted us to hypothesize that the delayed muscles relaxation observed in Romagnola cattle might also be the consequence of prolonged elevation in cytoplasmic free Ca^2+^ concentration. In order to validate whether a deficiency in sarcoplasmic reticulum Ca^2+^-ATPase activity might also underlies the pseudomyotonia in Romagnola cattle, we analyzed SERCA1 function in bovine skeletal muscles of affected and control cattle, respectively. The functional assay indicated that fast-twitch skeletal muscles of affected cattle showed a reduced activity, varying from 4% to 14% of controls (Figure 2A). These data are in agreement with reduced sarcoplasmic reticulum SERCA1 activity in other PMT affected cattle [3,4] and in human Brody's disease [6].

Sarcoplasmic reticulum microsomal fractions probed with anti-SERCA1 monoclonal antibodies showed that the amount of SERCA1 protein was decreased in the pathological samples with respect to control samples (Figure 2B). Although the SERCA1 staining was variable in PMT affected muscles, the reduction in SERCA1 protein density consistently correlated with the decrease in Ca^2+^-ATPase activity. Case 3 that showed the faintest SERCA1 band exhibited the most severe pathology, as demonstrated by active muscle damage and regeneration (Figure 1). Sarcoplasmic reticulum fractions from pathological and control samples were also probed with specific antibodies against protein markers of junctional (calcium release channel/ryanodine receptor 1) and non-junctional (sarcalumenin and its splice variant 53 kDa glycoprotein) sarcoplasmic reticulum membranes. As in PMT affected Chianina cattle [4], sarcoplasmic reticulum fractions from PMT affected Romagnola cattle did not differ in content of either junctional or non-junctional membrane markers, indicating that only SERCA1 is selectively affected.

### Genetics

**Figure 3:**
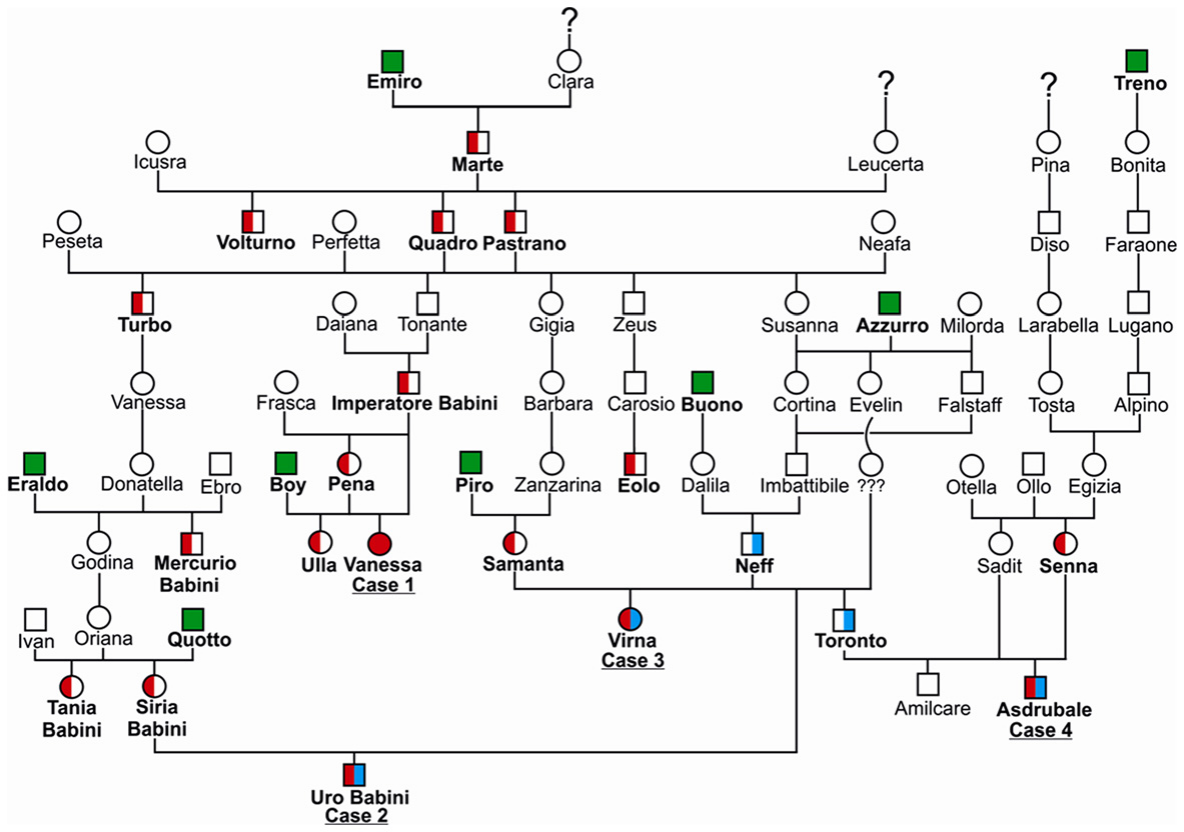
**Pedigree chart of the cattle family presented in this study.** The pseudomyotonia affected animals are shown as solid red symbol (case 1) or as symbols with filled shapes of red and blue (cases 2, 3, and 4). Healthy animals carrying the *ATP2A1* exon 8 variants (c.[632 G>T; 857 G>T]) are shown as red half-filled symbols. Carriers of the *ATP2A1* exon 6 mutation (c.491 G>A) are shown as blue half-filled symbols. Animals genotyped as non-carriers for both mutations are indicated as green squares. Open symbols represent healthy relatives with an unknown genotype. (Males are shown as squares and females as circles).

The parents of all available cases were healthy. Analysis of the available pedigree data revealed that two closely related artificial insemination sires (*Neff* and his son *Toronto*) had three PMT affected offspring among their progeny (Figure 3). A non-viable full sib of case 3 was aborted and therefore the phenotype of this animal remained unclear. An additional single PMT affected animal (case 1) was observed after a father daughter inbreeding mating (Figure 3). Pedigree records showed that the sire of case 1 (*Imperatore Babini*) is distantly related to the fathers of the other PMT affected animals. Under the assumption of recessive inheritance as described for PMT in other cattle breeds we searched for common ancestors of the four PMT cases. Three PMT affected animals (case 1, 2 and 3) could be traced back both on the maternal and the paternal path to a single common male ancestor (*Marte*) born in 1978 (Figure 3). For case 4 we established only a paternal relationship to this possible founder sire. Therefore, we concluded from the pedigree data that the causative mutation may have occurred in an unknown common ancestor. Due to missing records in the database we were not able to collect further information.

### ATP2A1 mutation analysis

**Figure 4:**
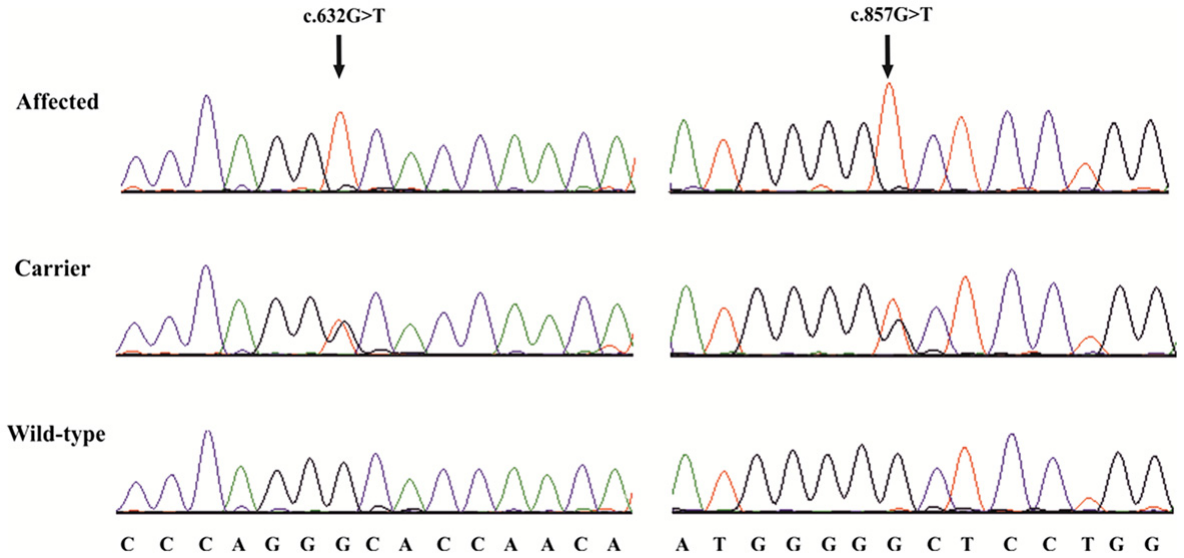
**Mutation analysis of the bovine *ATP2A1 *exon 8.** Sequence traces of genomic DNA of an affected, an obligate heterozygous carrier, and an unrelated control animal are shown. Arrows denote the positions of the variants. Numbering of nucleotides and codons is according to the open reading frame of the cDNA sequence (Acc. NM_001075767).

After we confirmed that the function of the SERCA1 pump is impaired in PMT affected Romagnola cattle and that the disease is recessively inherited, we performed a mutation analysis of *ATP2A1*. Mutation analysis in the four re-sequenced PMT affected animals revealed a total of 3 single nucleotide polymorphisms (SNPs) in comparison to *ATP2A1* of the cattle reference genome sequence (Table 1). Cases 2–4 were heterozygous for a missense SNP in exon 6 (c.491 G>A). This variant causes recessively inherited PMT in Chianina cattle [4,7]. In addition, the same three PMT affected animals were heterozygous for two SNPs in *ATP2A1* exon 8 (c.[632 G>T; 857 G>T]) (Figure 4). The PMT affected case 1 did not carry the exon 6 variant, but was homozygous for the two SNPs in exon 8 (Table 1). Taken together, the identified *ATP2A1* variants were associated with PMT under the assumption of a monogenic autosomal recessive inheritance (Figure 3). All 7 obligate carriers (parents of PMT affected offspring) were either heterozygous G/A for the exon 6 variant or heterozygous G/T for both exon 8 variants (Table 1 and Figure 3). All four dams of PMT cases carry both exon 8 variants in a heterozygous state. On the paternal side, only the sire of case 1 is heterozygous for the exon 8 variants, the two other sires of PMT affected animals are not carrying the exon 8 variants, but they are heterozygous carriers of the exon 6 variant (Table 1 and Figure 3). The mutant exon 8 allele was found in a total of 13 additional Romagnola cattle, which all were heterozygous for both SNPs. These presumed PMT carrier animals are closely related to the parents of the affected calves including the previously identified common male ancestor *Marte* (Figure 3). The exon 6 and both exon 8 variants are rare in the breed as they were absent from additional 192 healthy Romagnola controls. In addition, the mutant exon 8 allele was absent from 100 control cattle from 20 diverse breeds. Apart from the 3 PMT cases no further compound heterozygotes were detected for the exon 6 and exon 8 SNPs in the Romagnola control cohort. In conclusion, we observed an association between the occurrence of the disease and the presence of *ATP2A1* mutations. A single PMT case was homozygous for the exon 8 variants, the other 3 cases were compound heterozygotes with a paternally inherited exon 6 mutation and maternally inherited exon 8 mutations (see Table 1). As we always observed the two nucleotide substitutions in exon 8 together on the same haplotype, we speculate that these two substitutions arose simultaneously in a complex mutation event.

**Table 1:** ***ATP2A1 *genotypes of four pseudomyotonia affected Romagnola cattle and their direct relatives**

**Animal**	**Phenotype**	**exon 6 (c.491 G>A)**	**exon 8 (c.632 G>T)**	**exon 8 (c.857 G>T)**
**Case 1**	PMT	G/G	**T**/**T**	**T**/**T**
**Dam of case 1**	normal	G/G	G/**T**	G/**T**
**Sire of case1**	normal	G/G	G/**T**	G/**T**
**Case 2**	PMT	**A**/G	G/**T**	G/**T**
**Dam of case 2**	normal	G/G	G/**T**	G/**T**
**Sire of case 2 and 3**	normal	**A**/G	G/G	G/G
**Case 3**	PMT	**A**/G	G/**T**	G/**T**
**Dam of case 3**	normal	G/G	G/**T**	G/**T**
**Case 4**	PMT	**A**/G	G/**T**	G/**T**
**Dam of case 4**	normal	G/G	G/**T**	G/**T**
**Sire of case 4**	normal	**A**/G	G/G	G/G

Compound heterozygous mutations causing recessively inherited genetic diseases are well known in human genetics but not described commonly in purebred domestic animals. As recently shown for PMT in Chianina cattle, the spreading of a single founder mutation after intense use of closely related artificial insemination carrier sires typically explains outbreaks of genetic disease in cattle populations [9]. The origin of the mutation in the ancestors of both breeds seems not probable to the authors, so we assume that the most likely scenario for the presence of the exon 6 mutation in Romagnola cattle is an accidental introgression of a Chianina PMT carrier animal in the ancestry of *Neff* (Figure 3).

### Possible impact of the SERCA1 mutations

**Figure 5:**
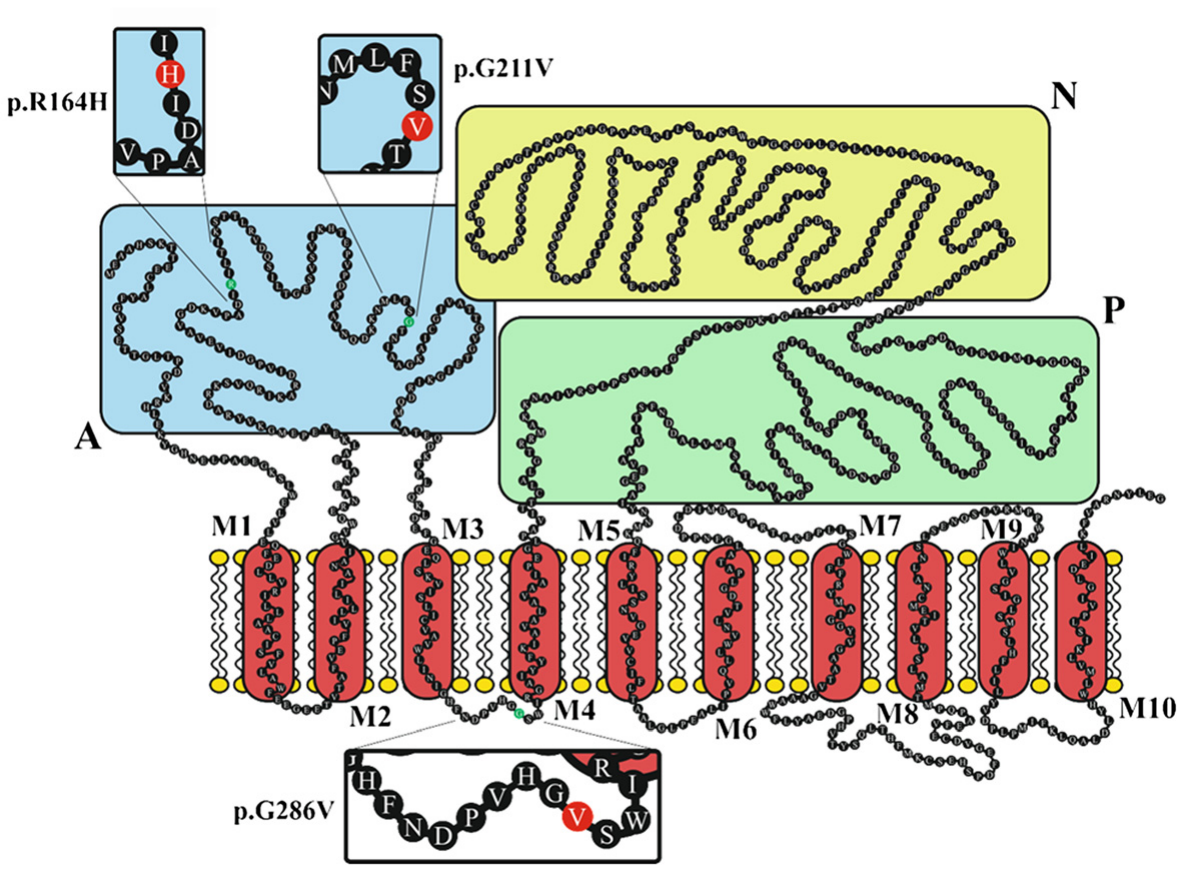
**Localization of the pseudomyotonia causing mutations in the bovine SERCA1 protein, according to Wuytak et al. [**[10]**].** A: actuator domain, P: phosphorylation domain; N: nucleotide binding domain. M1-10: transmembrane domain. The three mutant amino acid residues are shown in red and displayed in the insets.

**Figure 6:**
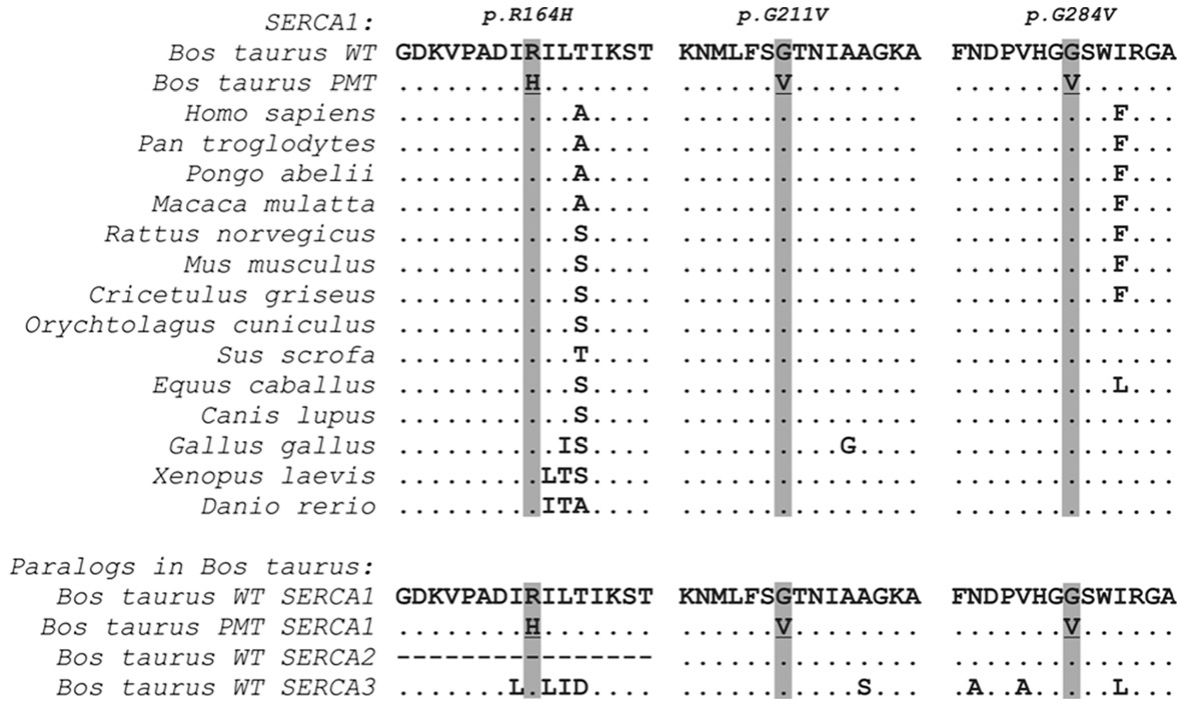
**Regions around the SERCA1 mutations.** Multiple sequence alignment of the SERCA1 protein in the region of the three different mutations demonstrates the conservation of residues across vertebrates. In addition, the mutant residues are conserved in all known bovine SERCA1 paralogs.

Genetic data showed that the PMT disorder is caused either by homozygosity or by compound heterozygosity of *ATP2A1* mutations. The previously reported exon 6 mutation leads to an amino acid exchange (p. Arg164His) within the actuator domain of the encoded SERCA1 protein [7]. The two newly identified exon 8 substitutions are predicted to result in non-conservative exchanges of glycine to valine at two different sites (p.[(Gly211Val; Gly284Val)]) of the SERCA1 protein sequence (Figure 5). The p. Gly211Val mutation also affects the mobile actuator domain. The p. Gly286Val mutation is situated in the direct proximity of the fourth transmembrane domain (Figure 5). Multiple protein sequence alignments show that the wild-type residues at the affected positions are conserved across all known SERCA1 orthologs in vertebrates including *Danio rerio* and *Xenopus laevis* (Figure 6).

We considered structure and function of SERCA1 to explain possible effects of the mutations causing PMT in Romagnola cattle. The SERCA1 protein consists of three cytosolic domains (see Figure 5) involved in the transmission of the major conformational changes [11]. The intracellular calcium pump is located in the sarcoplasmic reticulum and transfers Ca^2+^ from the cytosol of the cell to the lumen of the sarcoplasmic reticulum at the expense of ATP hydrolysis during muscle relaxation. The mechanism of the transport has been explained in term of a cycle characterized by two conformational states: E1 (Ca^2+^-bound form, high calcium affinity, protein works as a kinase phosphorylating Asp351) and E2 (Calcium released, Ca^2+^ affinity is low, protein works as a phosphatase) [12]. Dramatic changes in orientation of the domains in the transition from E1 to E2 were described [11,13]. In detail, the actuator domain is described as subjected to several rotations and to interact with phosphorylation and nucleotide binding domain [14]. Furthermore, there have to be movements of the helices in the transmembrane region to generate pathways for the entry or the release of the Ca^2+^ ions [15].

In PMT affected Romagnola cattle the side chain hydrogen atoms of glycines 211 and 286 are substituted by the much larger aliphatic hydrophobic side chain of valine, which tends to be buried in the center of protein, away from the aqueous environment. In both cases, the substitutions could have a role in disfavoring the proper folding of the protein. Recently, the crystal structure of the bovine SERCA1 has been reported [16]. The overall molecular model is very similar to that of the rabbit enzyme [13]. The PMT causing mutations in SERCA1 described here occur in the actuator domain of SERCA1 and in the lumenal loop where the similarity between the bovine and rabbit crystal structure is very high.

The putative consequences of the mutations were also evaluated with the help of several software programs. In particular, they predicted the mutation as highly damaging, and causing loss of a turn and the extension of a beta-sheet as a consequence of the p. Gly211Val mutation, and possible loss of stability near a transmembrane domain. This could be probably detrimental to the protein functioning considering the great interaction of the actuator domain with other parts of the protein and the numerous movements the domain is subjected to (see Additional file 2). The significantly reduced SERCA1 activity implicates a partial loss of function of these mutations. However, the PMT affected Romagnola animals showed no clinically obvious different disease signs compared to affected Chianina cattle. The impact of the mutations reported in this paper are not directly comparable with the p. Arg559Cys reported in Belgian Blue [2] since it consists in the substitution of a residue interacting directly with a nucleotide. Additionally, it is difficult to compare the effect of the mutations in cattle to the ones found in Brody’s disease, since the reported human causative mutations [17,18] affect other parts of the SERCA1 protein and are neither observed in non-human species [19].

## Conclusions

In pseudomyotonia affected animals the Ca^2+^-ATPase activity of sarcoplasmic reticulum membranes (SERCA1) was markedly decreased compared to control animals. We were able to successfully detect one known and one novel complex *ATP2A1* variant associated with the cases of pseudomyotonia. Some cases showed compound heterozygous genotypes. This study is, to the author’s knowledge, the first time that compound heterozygosity for different mutations in a single gene has been reported to cause genetic disorder in livestock. Furthermore, the knowledge of the new amino acid substitutions and their effect on the phenotype provides further insight into the function of the skeletal muscle SERCA1 calcium pump. Finally, selection against these mutations can now be used to eliminate the mutant alleles from the Romagnola breed.

## Methods

### Ethics statement

All animal work has been conducted according to the national and international guidelines for animal welfare. The cattle owner agreed that the samples can be used for our study. The data were obtained during diagnostic procedures that would have been carried out anyway. This is a very special situation in veterinary medicine. As the data are from client-owned cattle that underwent veterinary exams, there was no “animal experiment” according to the legal definitions in Italy.

### Animals

Blood and tissue samples were taken from the affected animals and from a total of seven parents (see Table 1 and Figure 3). Genomic DNA was isolated using the DNeasy Blood & Tissue Kit (Qiagen) according to the manufacturer’s protocol. In addition, archived DNA samples of 205 Romagnola bulls and 100 animals from 20 different cattle breeds (Angus (n = 5), Aubrac (n = 1), Belgian blue (n = 3), Blonde d’Aquitaine (n = 2), Brown Swiss (n = 10), Charolais (n = 5), Chianina (n = 10), Eringer (n = 5), Galloway (n = 2), Hereford (n = 3), Scotish Highland (n = 4), Holstein (n = 10), Jersey (n = 3), Limousin (n = 10), Montbéliarde (n = 4),, Piedmontese (n = 2), Pinzgauer (n = 5), Salers (n = 1), Simmentaler (n = 10), Tyrolean Grey (n = 5)) were used for genotyping the *ATP2A1* exon 8 variants.

### Histopatholgical examination

We obtained muscle biopsies of the semimembranosus muscle from case 2 and case 3 (Figure 3). Muscle samples were frozen in cold isopentane and sections (10 μm) were cut in a cryostat. Serial cryostat sections were stained by: Hematoxylin and Eosin (H&E), Gomori’s trichrome, periodic acid-Schiff (PAS), succinic dehydrogenase (SDH), cytochrome oxidase (COX), or were immunostained by incubating with polyclonal antibodies (dilution 1:200) to neonatal myosin heavy chain (MHC) isoform [4], followed by incubation with secondary antibody conjugated with peroxidase (Dako, Milano, Italy). Reaction was visualized with the Envision method (Dako). Images were acquired with a bright field microscope (Olympus Vanox AH-3, Japan), equipped with video camera and analyzed with image analysis software (Olympus DP-software-70).

### Biochemical analysis

A crude microsomal fraction enriched in content of sarcoplasmic reticulum membranes was isolated by differential centrifugation from muscle biopsies from semimembranosus muscle of three PMT affected Romagnola animals. Protein concentration was determined by the method of Lowry et al. [20]. The Ca^2+^-ATPase activity of sarcoplasmic reticulum microsomal fraction, has been measured at optimum pCa (pCa5), in the presence of Ca^2+^-ionophore A23187. Equal quantities (3 μg/lane) of crude sarcoplasmic reticulum microsomal fractions were separated by 5-10% SDS-PAGE and immunoblotting was performed as described before [16].

### Genealogical analysis

The pedigrees of all animals included in the study were evaluated on the basis of the database of the Italian beef cattle breeding organization [21].

### Genotyping

Sequencing of all 22 coding exons including flanking intronic sequences of *ATP2A1* was performed for all four cases as described before [7]. The PMT causing mutations in all the relatives and controls were genotyped by direct re-sequencing of two PCR products containing bovine *ATP2A1* exon 6 and exon 8, respectively.

### Protein sequence analysis

Multiple sequence alignment was performed with ClustalW [22]. Impact of the mutations on the protein stability and structure was predicted with the following software tools: PolyPhen2 [23]; NetTurnP [24]; NetSurfP [25]; the Chou&Fasman secondary structure prediction server CFSSP [26]; PoPMuSiC [27]; Phyre2 [28]. For transmembrane domain prediction we used THMM [29] and THMpred [30].

## Competing interests

The authors declare that they have no competing interests.

## Authors' contributions

LM, RS, and CD did the experimental work and drafted the manuscript. ST, RL and AG examined the affected animals. ST, TD, FM and AG provided samples. CD and AG supervised the work and performed manuscript editing. All authors read and approved the final manuscript.

## Supplementary Material

Additional file 1 **PMT affected Romagnola cattle.** Two young Romagnola animals during muscle exercise showing typical signs of pseudomyotonia. (MPEG 9223 kb) Click here for file

Additional file 2 **Predicted consequences of bovine SERCA1 mutations.** NetSurfP and NetTurnP predicted the loss of a turn and the extension of a beta-sheet as a consequence of the p. Gly211Val mutation. This could be probably damaging considering the great interaction of the actuator domain with other parts of the protein and the numerous movements the domain is subjected to. PolyPhen2 and PoPMusiC classified the p. Gly211Val mutation as potentially damaging with the maximum possible score. The consequences of the p. Gly286Val mutation are predicted as being slightly less dramatic. The results from the transmembrane domain prediction tools are less clear-cut and identical for both new SERCA1 mutations. It is interesting to note that the residue 211 and 286 mutations are listed in general as being more fatal than the p. Arg164His. As reported [15,16], the second exon 8 mutation lies in a conserved part between M3 and M4 – we could infer hence that the part of the protein near M3 is disturbed in its stability leading to the prediction of a less likely M3 domain. The significantly reduced SERCA1 activity implicates a partial loss of function of these mutations. However, the PMT affected Romagnola animals showed no clinically obvious different disease signs compared to affected Chianina cattle. The software has been used to predict the effect of the mutation found in Belgian Blue and reported by [2]. No effect on turns or transmembrane domain is predicted (as reported by the authors who indicate the mutation as affecting a nucleotide binding domain) but is generally predicted as destructive by Poliphen and PoPMusiC (“highly damaging” by the latter). In addition, Phyre2 predicts an increase of chaos in the position. Click here for file
