# Supplementary material for: Pseudomyotonia in Romagnola cattle caused by novel ATP2A1 mutations
Source: BMC Vet Res. 2012 Oct 9;8:186. doi: 10.1186/1746-6148-8-186 (PMC3545862; doi:10.1186/1746-6148-8-186)
Supplement: Additional file 2 — Predicted consequences of bovine SERCA1 mutations. NetSurfP and NetTurnP predicted the loss of a turn and the extension of a beta-sheet as a consequence of the p. Gly211Val mutation. This could be probably damaging considering the great interaction of the actuator domain with other parts of the protein and the numerous movements the domain is subjected to. PolyPhen2 and PoPMusiC classified the p. Gly211Val mutation as potentially damaging with the maximum possible score. The consequences of the p. Gly286Val mutation are predicted as being slightly less dramatic. The results from the transmembrane domain prediction tools are less clear-cut and identical for both new SERCA1 mutations. It is interesting to note that the residue 211 and 286 mutations are listed in general as being more fatal than the p. Arg164His. As reported [15,16], the second exon 8 mutation lies in a conserved part between M3 and M4 – we could infer hence that the part of the protein near M3 is disturbed in its stability leading to the prediction of a less likely M3 domain. The significantly reduced SERCA1 activity implicates a partial loss of function of these mutations. However, the PMT affected Romagnola animals showed no clinically obvious different disease signs compared to affected Chianina cattle. The software has been used to predict the effect of the mutation found in Belgian Blue and reported by [2]. No effect on turns or transmembrane domain is predicted (as reported by the authors who indicate the mutation as affecting a nucleotide binding domain) but is generally predicted as destructive by Poliphen and PoPMusiC (“highly damaging” by the latter). In addition, Phyre2 predicts an increase of chaos in the position. [file 1746-6148-8-186-S2.pdf]

Additional file 2 Predicted consequences of bovine SERCA1 mutations.

Software	Prediction	p.Arg164His (Chianina)	p.Gly211Val (Romagnola)	p.Gly286Val (Romagnola)	p.Arg559Cys (Belgian Blue)
**Polyphen 2**	impact of an amino acid substitution on the structure and function of a protein	possibly damaging (score of 0.903)	probably damaging (score 1.000)	probably damaging (score 0.994)	probably damaging (score 1.000)
**Net TurnP**	possible changes in the formation of Beta-turns	*	predicted beta turn not formed in mutant protein	*	*
**NetSurfP**	surface accessibility and secondary structure	*	*	changes from buried to exposed	*
**CFSSP**	secondary structure of protein from the amino acid sequence	*	elimination of a turn (two beta-sheets joined)	elimination of a turn	*
**PoPMuSiC**	changes in stability of a given protein or peptide under single-site mutations	neutral mutation	destabilizing mutation	destabilizing mutation	destabilizing mutation
**Phyre 2**	secondary structure and disorder	*	elimination of a turn (two beta-sheets joined)	small alpha helix extended (1 residue)	increased disorder in 460-472 region
**TMHMM / THMpred**	transmembrane helices and topology of proteins	*	probability decrease in prediction of a transmembrane domain in the M3 subunit by ca. 20%	probability decrease in prediction of a transmembrane domain in the M3 subunit by ca. 20%	*

* no change predicted
